# Myokine Responses to Exercise in a Rat Model of Low/High Adaptive Potential

**DOI:** 10.3389/fendo.2021.645881

**Published:** 2021-06-09

**Authors:** Wesam F. Farrash, Bethan E. Phillips, Steven L. Britton, Nathan Qi, Lauren G. Koch, Daniel J. Wilkinson, Ken Smith, Philip J. Atherton

**Affiliations:** ^1^ Medical Research Council (MRC)-Versus Arthritis Centre for Musculoskeletal Ageing Research, Clinical, Metabolic and Molecular Physiology, University of Nottingham, Derby, United Kingdom; ^2^ Applied Medical Sciences, Umm Al-Qura University, Makkah, Saudi Arabia; ^3^ National Institution for Health Research (NIHR) Nottingham Biomedical Research Centre, School of Medicine, University of Nottingham, Derby, United Kingdom; ^4^ Department of Anesthesiology, University of Michigan, Ann Arbor, MI, United States; ^5^ Department of Molecular and integrative physiology, University of Michigan, Ann Arbor, MI, United States; ^6^ Department of Physiology and Pharmacology, University of Toledo, Toledo, OH, United States

**Keywords:** LRT-HRT, myokines, muscle, heterogeneity, adaptive response, exercise

## Abstract

**Introduction:**

Assuming myokines underlie some of the health benefits of exercise, we hypothesised that ‘high responder trainer’ (HRT) rats would exhibit distinct myokine profiles to ‘low responder trainers’ (LRT), reflecting distinct health and adaptive traits.

**Methods:**

Blood was collected from LRT and HRT (N=8) rats at baseline (BL), immediately (0h), 1h, and 3h after running; repeated after 3-wks training. Myokines were analysed by ELISA (i.e. BDNF/Fractalkine/SPARC/Irisin/FGF21/Musclin/IL-6).

**Results:**

At baseline, Musclin (LRT: 84 ± 24 vs HRT: 26 ± 3 pg/ml, *P=0.05*) and FGF21 (LRT: 133 ± 34 vs HRT: 63.5 ± 13 pg/ml, *P=0.08*) were higher in LRT than HRT. Training increased Musclin in HRT (26 ± 3 to 54 ± 9 pg/ml, *P<0.05*) and decreased FGF21 in LRT (133 ± 34 to 60 ± 28 pg/ml, *P<0.05*). Training increased SPARC (LRT: 0.8 ± 0.1 to 2.1 ± 0.6 ng/ml, *P<0.05*; HRT: 0.7 ± 0.06 to 1.8 ± 0.3 ng/ml, *P=0.06*) and Irisin (LRT 0.62 ± 0.1 to 2.6 ± 0.4 ng/ml, *P<0.01*; HRT 0.53 ± 0.1 to 2.8 ± 0.7 ng/ml, *P<0.01*) while decreasing BDNF (LRT: 2747 ± 293 to 1081 ± 330 pg/ml, *P<0.01*; HRT: 1976 ± 328 to 797 ± 160 pg/ml, *P<0.05*). Acute exercise response of Musclin (AUC) was higher in LRT vs HRT (306 ± 74 vs. 88 ± 12 pg/ml×3h^-1^, *P<0.01*) and elevated in HRT after training (221 ± 31 pg/ml×3h^-1^, *P<0.01*). Training elevated SPARC (LRT: 2.4 ± 0.1 to 7.7 ± 1.3 ng/ml×3h^-1^, *P<0.05*; HRT: 2.5 ± 0.13 to 11.2 ± 2.2 ng/ml×3h^-1^, *P<0.001*) and Irisin (LRT: 1.34 ± 0.3 to 9.6 ± 1.7 ng/ml×3h^-1^, *P<0.001*; HRT: 1.5 ± 0.5 to 12.1 ± 1.9 ng/ml×3h^-1^, *P<0.0001*).

**Conclusion:**

Exercise training alters how myokines are secreted in response to acute exercise. Myokine responses were not robustly linked to adaptive potential in aerobic capacity, making them an unlikely regulator of adaptive traits.

## Introduction

Physiological adaptations to exercise training are highly variable within individuals. For instance, individual responses in VO_2_ max vary from no change, to improvements of 50% (1) and 58% (2) in young and elderly individuals, respectively. At the extremes of these responses there are individuals who show large improvements; “high responders”, and others who show no improvement or even a reduced performance; “low responders” (3). A major challenge is to understand the biology underpinning this phenomenon. At its core, exercise capacity is under the control of: i) intrinsic levels of fitness of an organism, and ii) extrinsic factors that manifest in response to training (4). The intrinsic factors are well-studied in large cohort studies e.g. the HERITAGE family studies (5). On the other hand, little is known about the extrinsic elemental role in regulating exercise capacity. Therefore, an animal model was developed, *via* selective out-breeding, wherein rats were cross-bred according to their adaptive performance, or lack of, i.e. maximal running distance, during a running to exhaustion treadmill test. Therein followed “low” and “high” trainability offspring from a heterogeneous rat population (N/NIH) (4). These so-called low response trainer (LRT) and high response trainer (HRT) animals exhibited similar exercise capacities (running a similar distance), at baseline; however, after 8-wks of endurance training, HRT animals were able to run 200 meters more, while LRT animals exhibited a decline in running capacity by 65 meters (4).

The multi-generational phenotype of the LRT/HRT animals has been extensively studied. Training capacity, assessed using VO_2_ max, is a strong indicator of morbidity and survivability (6) e.g. with improvements in VO_2_ max following aerobic exercise training being linked with the mean life span in both animals (7, 8) and humans (9, 10). In addition, LRT animals were prone to increased metabolic dysfunction e.g. insulin resistance, obesity and reduced muscle angiogenesis (11). It is well established that exercise training initiates a plethora of beneficial adaptive responses. In muscle, for example, aerobic and resistance exercise training improves skeletal muscle endurance and strength respectively, and both modes of training enhance skeletal muscle metabolism (uptake and utilisation) of glucose and fat (12, 13). Thus, exercise training is associated with reduced risk of developing chronic diseases such as obesity, type 2 diabetes (T2D), and cardiovascular disease (14). Yet the upstream drivers (i.e. triggers of signalling pathways regulating established adaptive processes) of exercise induced adaptations at the level of muscle and at a trans-organ and whole-body level, are complex. One hypothesis, that has received much recent attention, is the notion that muscle produces “exercise factors or myokines” in response to contractile activity (i.e. exercise). These myokines are released from muscle and are purported to act locally, in an auto/paracrine fashion, or distally in an endocrine fashion on other organs and tissues. Reflecting this, a large number of ‘putative’ myokines have been shown to be expressed in, and secreted from muscle in response to exercise (15, 16). The implication of this, is that myokines construct a communication axis within and between skeletal muscle, remote tissues, and organs like liver, adipose tissue, heart, brain and the vasculature, to exert their effects in an endocrine fashion. For instance, circulating Irisin enhances the browning of subcutaneous white adipose tissue (17), circulating interleukin 6 (IL-6) improves hepatic glucose release (18), Secreted Protein Acidic and Rich in Cysteine (SPARC) increases bone formation, and Brain-derived neurotrophic factor (BDNF) improves cognitive ability (19). In addition, a number of these myokines (e.g., IL-6, Irisin, SPARC, and BDNF) also demonstrate paracrine/autocrine effects on skeletal muscle; improving glucose uptake and mitochondrial respiration, which has been linked to improved insulin sensitivity. Furthermore, Irisin, SPARC, IL-15, and leukaemia inhibitory factors (LIF) have been suggested to encourage muscle hypertrophy (16, 20, 21). Hundreds of myokines have been proposed, but only a very few have been more robustly characterized and their response to exercise and impact on biological function demonstrated (22). We investigated a representative collection of established myokines in this study.

To further define the role of myokines in exercise adaptation, we adopted the LRT/HRT rat model of distinct responder status in adaption to exercise. We hypothesised that, reflecting established differences in adaptive potential and metabolic health, HRT animals would show greater myokine concentrations at baseline, in response to acute exercise, and that this would be sustained following a running training intervention.

## Methods

### Experiment Design and Ethics

**Figure 1:**
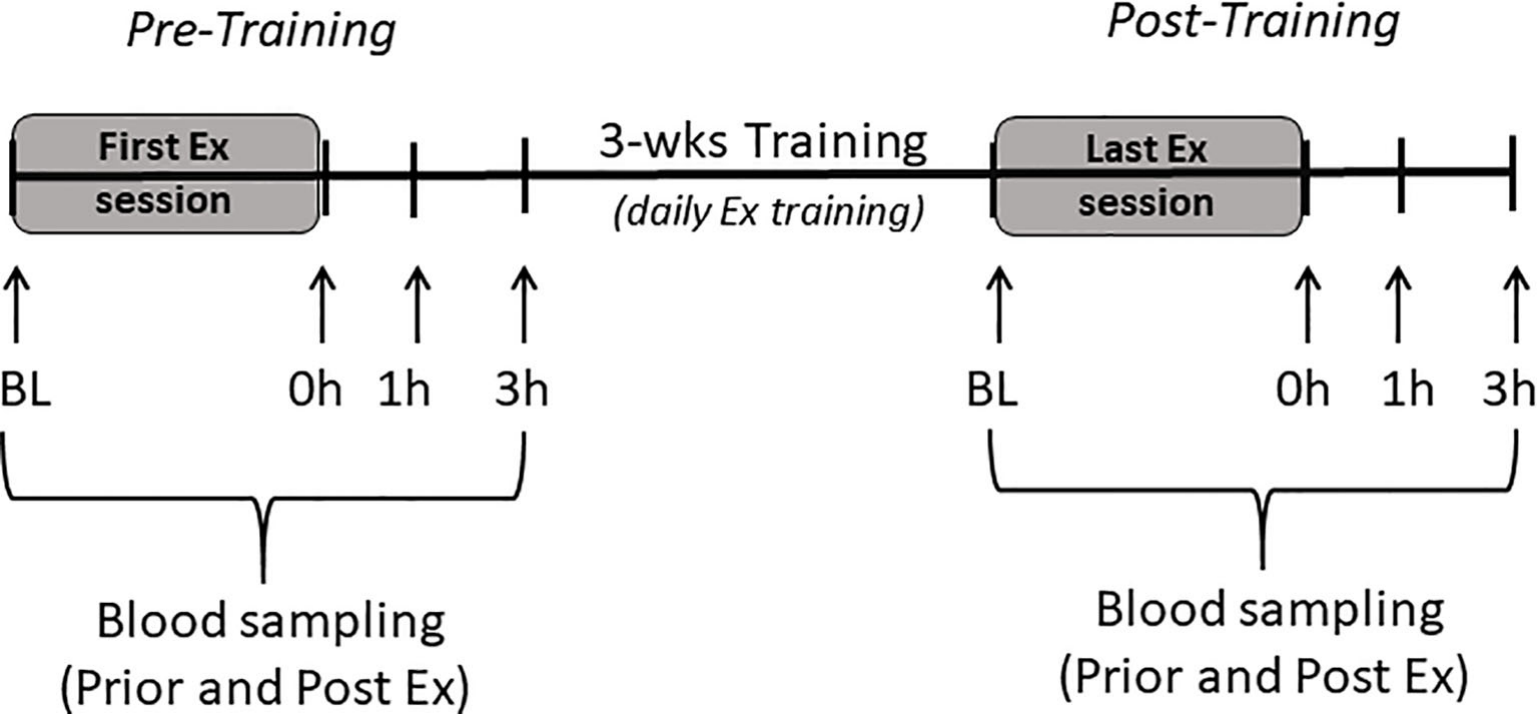
Study scheme. Baseline (BL), immediate (0h), one hour (1h), and 3 hours (3h) after exercise session (Ex).

**Figure 2:**
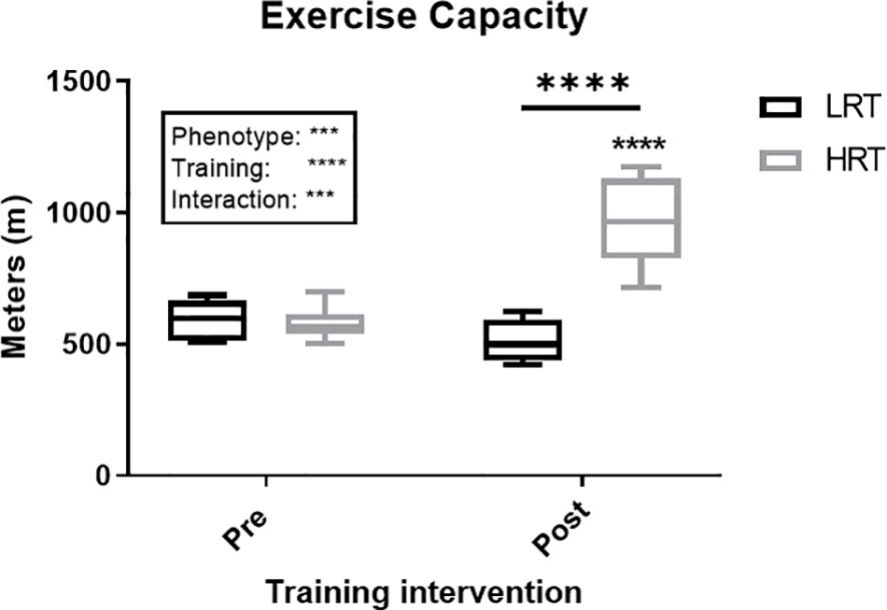
Change in exercise capacity (meters) in low response trainers (LRT) and high response trainers (HRT). Values are means±SD. Analysis via two-way ANOVA with Sidak’s post hoc test. Main effects are shown in the text box. ***P<0.001, ****P<0.0001. Non-underlined symbols represent within group differences. Underlined symbols represent between group differences.

All procedures were carried out in accordance with the Institute for Laboratory Animals Research Guide for Care and Use of Laboratory Animals and in compliance with guidelines as reviewed by the University Committee on Use and Care of Animals at University of Michigan. All animals were housed in pathogen-free facilities under a 12:12-h light-dark cycle at room temperature of 22°C. All animals were fed rodents pellet diet (diet #5001; Purina Mills, Richmond, IN) and water was provided ad libitum. LRT/HRT animals were studied; these animals were developed by selective breeding and maintained by Koch and Britton at the University of Michigan (4). After 2-wks of acclimatization to surrounding environment, a total of 16 female rats from the 24^th^ generation (n=8 LRT, and n=8 HRT) performed an acute bout of exercise, which included 30 min of treadmill running (15% incline) at moderated speed (17m/min) and targeted 60-70% of VO_2_ max. Tail vein blood samples were collected prior to the exercise training bout, immediately following and again at 1h and 3h post exercise (pre-training). Animals then underwent a 3-wk training intervention performing daily exercise sessions (as described above). During the last exercise session, blood samples were collected as during the first acute bout of exercise (post-training) (**Figure 1**). Animals had an exercise capacity test (running distance) before and after the training intervention to calculate their exercise response (**Figure 2**). Changes in animal’s body weight were also reported before and after the intervention (**Supplementary Figure 1**).

### Multiplex ELISA

A Multiplex ELISA (Rat Myokine Magnetic Beads Panel, cat # RMYOMAG-88K, MeRCK) was used to quantitate multiple-analytes from the same sample. The kit intra and inter precision are <10% and <20% respectively. The sensitivity with the minimum detection level of target myokines are listed in **Table 1 (Supplementary Data)**. The protocol followed manufacturer instructions. Antibody-immobilized beads for the different analytes were sonicated, vortexed, and then transferred together to a bead mix bottle. Quality controls, washing buffers, serum matrix, and standards were prepared with ddH_2_O and a 7-point standard curve constructed. Each well was washed with 200µl of washing buffer then decanted. 25µl of standards, controls and samples were added to designated wells. Before adding 25µl of mixed beads, serum matrix was added to standards and controls (to mimic the sample media). The plate was incubated in a dark cold room at 4°C for 16 hours with gentle agitation. The content of each well was removed, and the plate was washed 3x with 200µl of washing buffer, using a handheld magnet to keep the beads in the wells during decanting. 25µl of detection antibody was added per well and incubated for 1 hour at room temperature with gentle agitation, before 25µl of streptavidin/HRP was added and incubated for 30 mins. Contents were removed and the plate was washed 3x, before 150µl of sheath fluid was pipetted into each well. The plate was then read on the MAGPI*X*, (powered by Luminex *X*MAP technology, MeRCK). Myokines concentrations were calculated with reference to standard curve.

### IL-6 ELISA Kit

A high-sensitivity IL-6 ELISA kit was performed in accordance to the manufacturer’s instructions (BMS625, Invitrogen; Thermo Fisher Scientific, Inc.). In brief, washing buffer, assay buffer, and standards were prepared and reconstituted with ddH_2_O and serial dilutions of the standard curve were externally prepared using assay buffer. A pre-coated 96 micro-well plate was used and read at 450nm. Results were calculated according to the best-fit standard curve.

### Statistical Analysis

To determine the difference between and within groups a two-way ANOVA was used. Further, we used Sidak’s *post hoc* testing for multiple comparisons. Data are presented as mean ± SD. A *P* value <0.05 was considered significant.

## Results

Of the 12 Myokines included in the Multiplex ELISA kit, only 6 were detectable in the plasma: Irisin, BDNF, SPARC, Fractalkine, Fibroblast growth factor 21 (FGF21) and Musclin, with Myostatin, IL-15, Follistatin-like protein-1 (FSTL-1), Erythropoietin and LIF below the limits of detection.

**Figure 3:**
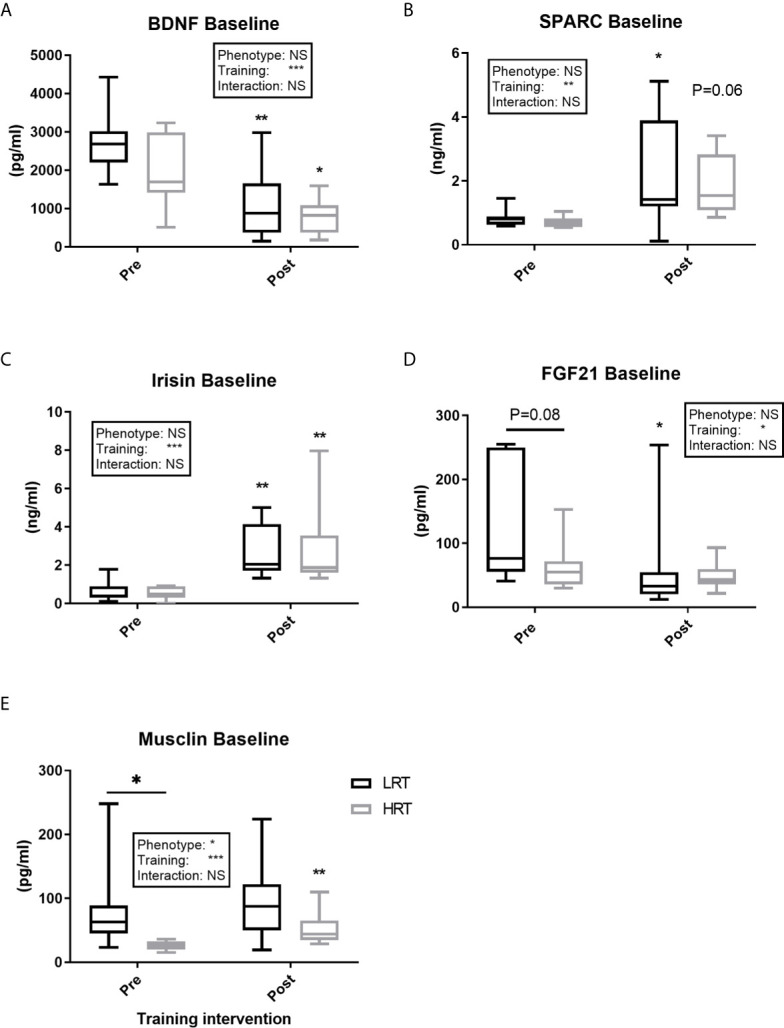
Baseline concentrations of plasma myokines pre and post exercise training. **(A)** Brain-derived neutrophic factor (BDNF); **(B)** Secreted protein acidic and rich in cysteine (SPARC); **(C)** Irisin; **(D)** Fibroblast growth factor 21 (FGF21); **(E)** Musclin. Values are means±SD. Analysis via two-way ANOVA with Sidak’s post hoc test. Main effects are shown in the text box. *P<0.05, **P<0.01, ***P<0.001, NS, Not Significant. Non-underlined symbols represent within group differences. Underlined symbols represent between group differences.

**Figure 4:**
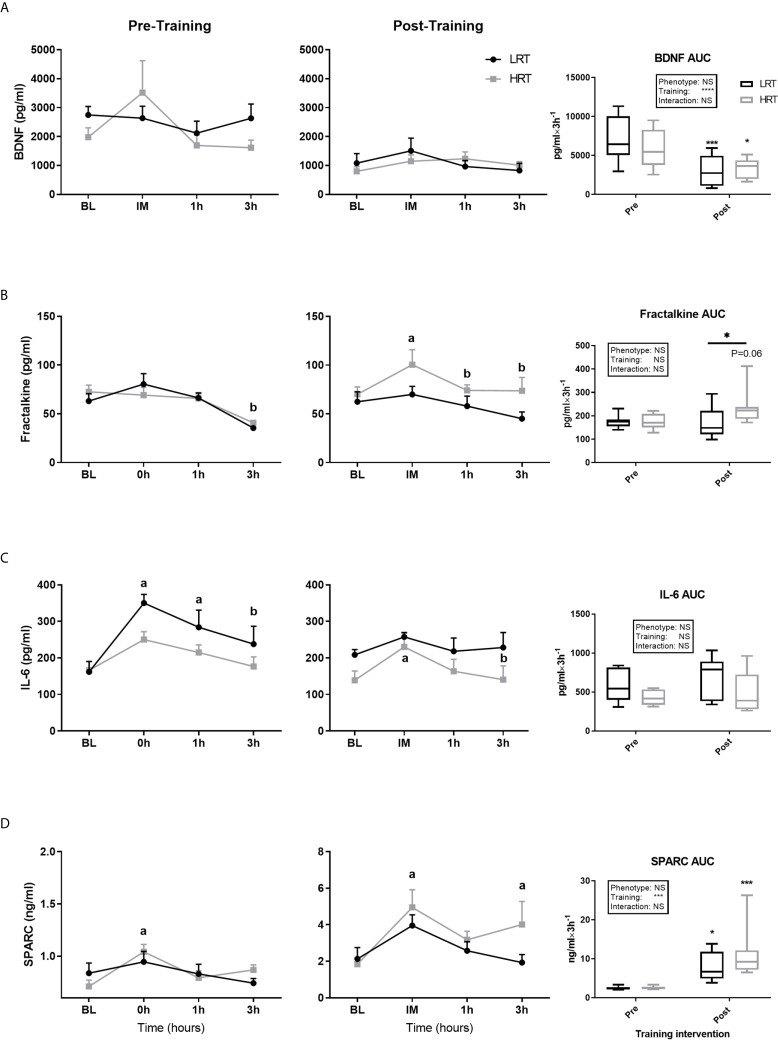
Temporal change in concentration of plasma myokines and area under the curve (AUC) before and after exercise training. **(A)** Brain-derived neutrophic factor (BDNF); **(B)** Fractalkine; **(C)** Interlukin-6 (IL-6); **(D)** Secreted protein acidic and rich in cysteine (SPARC); Baseline (BL). Values are means±SD. Analysis via two-way ANOVA with Sidak’s post hoc test. Main effects are shown in the text box. *P<0.05, ***P<0.001, NS, Not Significant. a=significant change from BL. b= significant change from 0h. Non-underlined symbols represent within group differences. Underlined symbols represent between groups differ.

BDNF concentrations at baseline, pre and post training, were similar between the LRT and HRT groups. However, the training intervention reduced the baseline concentrations significantly in both animals (LRT: 2747 ± 293 to 1081 ± 330 pg/ml, *P<0.01*; HRT: 1976 ± 328 to 797 ± 160 pg/ml, *P<0.05*) (**Figure 3A**). Similarly, the acute temporal response to exercise, expressed as area under the curve (AUC), did not show any significant differences between the groups pre or post training. However, the total AUC was lower in both groups after the training intervention (LRT: 7127 ± 1015 to 3028 ± 681 pg/ml×3h^-1^, *P<0.001*; HRT: 5918 ± 872 to 3429 ± 448 pg/ml×3h^-1^, *P=0.01*) (**Figure 4A**).

Fractalkine baseline concentrations were not different between the LRT and HRT groups pre or post training and there was no impact of training on these concentrations. Regarding the acute temporal response to exercise, after the first exercise session, Fractalkine concentrations dropped at 3 hours in both groups (LRT: BL, 63 ± 7.7 to 3h, 35.4 ± 2.7 pg/ml, *P<0.05*; HRT: BL, 72.5 ± 6.7 to 3h, 40.8 ± 2.3 pg/ml, *P<0.01*). Conversely, in response to the last training session, the HRT group displayed an immediate increase in Fractalkine (BL, 70 ± 7.5 to 0h, 100 ± 15.4 pg/ml, *P<0.05*) that returned to baseline by 1 hour [1h, 74 ± 5.8 pg/ml, *P=0.05* (and 3h, 73.6 ± 13.6 pg/ml, *P=0.05*)], with no changes observed in the LRT group. In addition, despite AUC data showing no significant impact of phenotype or training on the temporal response of Fractalkine to exercise, there was a tendency towards an increased post-training AUC in the HRT group only (174 ± 28 vs. 234 ± 26 pg/ml×3h^-1^, *P=0.06*), resulting in a post-training AUC that was significantly higher in the HRT group compared to LRT (166 ± 25 pg/ml×3h^-1^, *P=0.05*) (**Figure 4B**). Of note, the percentage of change (%Δ) in Fractalkine AUC over the training intervention correlated with %Δ in exercise capacity (r=0.63, *P<0.001*), i.e. the improvement in Fractalkine temporal response to acute exercise is in line with the improvement in running distance.

IL-6 baseline concentrations were similar between the groups before and after training, with no effect of training on baseline levels in either group. Regarding the temporal response to acute exercise before training, the LRT group showed an immediate increase in IL-6 from baseline (BL, 161 ± 28 to 0h, 350 ± 23 pg/ml, *P<0.0001*), which was sustained at 1 hour (1h, 283 ± 47 pg/ml, *P<0.05*) but returned towards baseline by 3 hours (3h, 237 ± 48, *P<0.05* vs. 0h). Conversely, before training the HRT animals showed no acute IL-6 response to acute exercise. After training, LRT animals showed no acute changes to exercise. However, in HRT animals there was an initial increase (BL, 138 ± 25 to 0h, 230 ± 37 pg/ml, *P<0.05*), which returned to baseline by 3 hours (3h, 140 ± 37 pg/ml, *P=0.05*). Despite these within group observations, there was no effect of phenotype or training on IL-6 temporal responses to exercise (**Figure 4C**).

Baseline concentrations of SPARC were similar across the groups before and after training, with a significant training-induced increase in baseline levels in the LRT group (0.83 ± 0.09 to 2.1 ± 0.6 ng/ml, *P<0.05*) and trend for an increase in the HRT group (0.70 ± 0.06 to 1.8 ± 0.3 ng/ml, *P=0.06*) (**Figure 3B**). Coupled to the increase in baseline SPARC after training, the AUC reflecting response to acute exercise also increased in both groups after training (LRT: 2.4 ± 0.1 to 7.7 ± 1.3 ng/ml×3h^-1^, *P<0.05*; HRT: 2.5 ± 0.13 to 11.2 ± 2.2 ng/ml×3h^-1^, *P<0.001*). Despite the similarity in AUC responses between the groups, only in the HRT animals was SPARC elevated immediately post exercise during both the first (BL, 0.7 ± 0.06 to 0h, 1.0 ± 0.07 ng/ml *P<0.05*) and last (BL, 1.8 ± 0.3 to 0h, 5 ± 0.9 ng/ml *P<0.01*) training session, and remained elevated at 3 hours in the last training session only (3h, 4.0 ± 1.2 ng/ml *P<0.05*) (**Figure 4D**). No time-point specific increases were observed in the LRT group before or after training.

**Figure 5:**
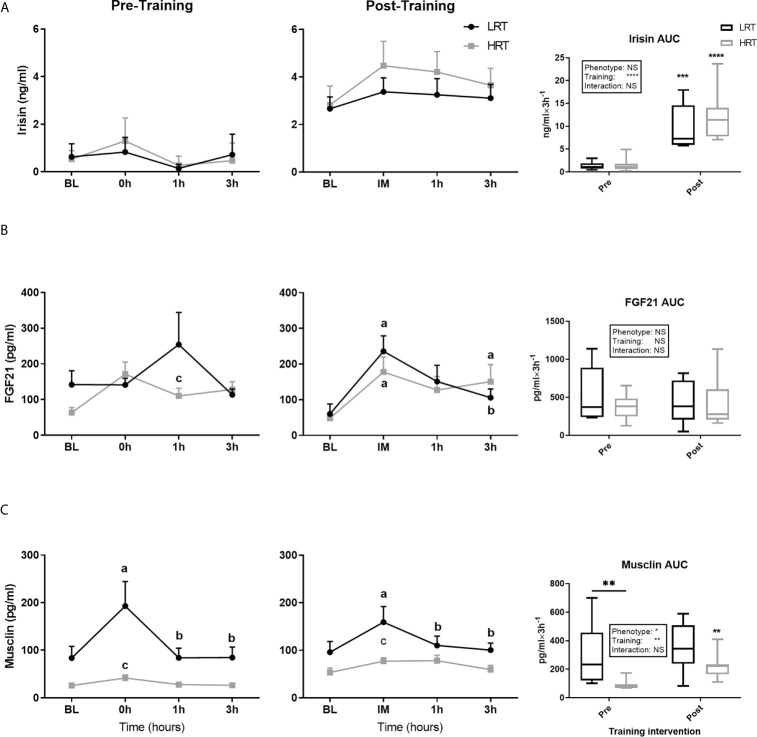
Temporal change in concentration of plasma myokines and area under the curve (AUC) before and after exercise training. **(A)** Irisin **(B)** Fibroblast growth factors 21 (FGF21). **(C)** Musclin. Baseline (BL). Values are means±SD. Analysis via two-way ANOVA with Sidak’s post hoc test. Main effects are shown in the text box. *P≤0.05, **P<0.01, ***P<0.001, ****P<0.0001, NS, Not Significant. a= significant change from BL. b= significant change from 0h. c. significant change across groups. Non-underlined symbols represent within group differences. Underlined symbols represent between group differences.

Irisin baseline concentrations were not different between the groups pre or post training, with training eliciting a similar increase in baseline concentrations in both groups (LRT: 0.62 ± 0.1 to 2.6 ± 0.4 ng/ml, *P<0.01*; HRT: 0.53 ± 0.1 to. 2.8 ± 0.7 ng/ml, *P<0.01*) (**Figure 3C**). The temporal response to acute exercise was similar in both groups before and after training, with a clear increase after training in the AUC of both groups (LRT: 1.3 ± 0.3 to 9.6 ± 1.7 ng/ml×3h^-1^, *P<0.001*; HRT: 1.5 ± 0.5 to 12.1 ± 1.9 ng/ml×3h^-1^ *P<0.0001*) (**Figure 5A**).

FGF21 baseline concentrations tended to be higher in LRT than HRT animals before training (133 ± 34 vs. 63.5 ± 13 pg/ml, *P=0.08*), although training decreased FGF21 baseline levels in the LRT group only (60 ± 28 pg/ml, *P<0.05*) such that there were no differences between the groups after training (**Figure 3D**). Temporal responses to acute exercise were similar between the groups both pre and post training, despite a higher pre-training concentration at 1 hour after exercise in the LRT group (254 ± 90 vs. 110 ± 21 pg/ml, *P<0.05*). In the last training session, both groups showed immediate increases from baseline (LRT: BL, 60 ± 28 to 0h, 235 ± 43 pg/ml, *P<0.0001*; HRT: BL, 48 ± 7 to 0h 177 ± 41 pg/ml, *P<0.01*), an elevation that remained present in the HRT group only [3h, 150 ± 47 pg/ml, *P<0.05*) and dropped in LRT (LRT: 3h, 105 ± 24 pg/ml, *P<0.01*)] (**Figure 5B**).

Before training Musclin concentrations at baseline were significantly higher in the LRT than HRT group (83 ± 24 vs. 25 ± 2 pg/ml, *P=0.05*). However, training elicited a significant increase in baseline concentrations in HRT animals only (53.6 ± 9.2 pg/ml, *P<0.01*) such that the baseline concentrations of the two groups were not significantly different after exercise (**Figure 3E**). Before training the response to acute exercise was higher in the LRT than HRT group (306 ± 74 vs. HRT 88 ± 12 pg/ml×3h^-1^, *P<0.01*). Further, and similar to the changes seen in baseline concentrations, training significantly increased the response to acute exercise in the HRT group only (221 ± 31 pg/ml×3h^-1^, *P<0.01*). As such, the response to acute exercise was not significantly different between the groups after training. Following acute exercise, both before and after training, a significant increase in Musclin was observed only in the LRT group, and only at the time-point immediately after exercise (pre-training: BL, 83.5 ± 24 to 0h, 192 ± 51 pg/ml, *P<0.0001*; post-training: BL, 95.9 ± 22 to 0h 159 ± 32 pg/ml, *P<0.0001*). As a result of these increases and higher baseline concentrations before training in the LRT group, Musclin concentrations immediately post-acute exercise were significantly higher in the LRT than HRT group both before (192 ± 51 vs. 41.6 ± 6.5 pg/ml, *P<0.0001*) and after (159 ± 32 vs. 77.2 ± 6.9 pg/ml, *P<0.01*) training (**Figure 5C**). In addition, the %Δ of Musclin AUC tended to correlate with %Δ of exercise capacity (r=0.47, *P=0.06*). While, %Δ of Musclin baseline and AUC negatively correlated with %Δ of animals body weight (r=-0.59, *P<0.05*, r=-0.63*, P<0.01*, respectively).

## Discussion

To gain further understanding of the potential role of myokines in underlying aspects of variation in adaptive response to exercise training (training capacity), we employed an experimental rat model of two divergent traits (LRT/HRT). LRT and HRT share a similar exercise capacity (running distance) at baseline, but HRT animals show a 54% improvement in running distance in response to chronic treadmill training, whereas LRT animals fail to respond (4). In addition, LRT animals have been reported as being prone to developing metabolic disorders such as insulin resistance and adiposity at a young age (11). However, while we show that exercise-induces, and training alters myokine responses, few consistent links to phenotype manifested.

### Baseline Differences in Resting Plasma Myokines Levels

Baseline myokine levels have been linked to human health and body composition. For example, FGF21 is elevated in obese individuals and those with T2D (23), and in mitochondrial myopathy and metabolic stress conditions (24). Similarly, BMI, fasting insulin, HOMA-IR, triglyceride concentration, and percentage fat (of whole-body mass) have been positively correlated to plasma levels of SPARC in humans (25, 26). Therefore, given that the metabolic health of LRT animals is considered “poorer” than HRT in term of insulin resistance and obesity (11), we hypothesised that this would manifest in the basal concentration of plasma myokines. In line with this, our observation of elevated FGF21 and Musclin in LRT animals at baseline (i.e. pre-training) somewhat supports previous reports of higher concentrations of FGF21 (23) and Musclin (27, 28) in diabetic and obese subjects (23, 27, 28). Elevated Musclin has previously been positively linked to high fasting glucose, triglycerides, HOMA-IR, and serum insulin (27, 28), concurrent with the LRT ‘metabolic condition’ (11). These high baseline levels may indicate a protective role of myokines against metabolic stress, as FGF21 administration has been shown to improve insulin sensitivity, plasma glucose, and triglycerides in diabetic mice (29). However, elevated baseline levels of myokines may also be a result of the development of “myokine resistance”; a phenomena similar to what happens with insulin, and that has been reported with FGF21 in liver and fat (30).

Upon investigating the impact of 3-wks exercise (running) training on baseline myokine profiles, we found that in general, alterations in baseline circulating myokines did not explain gross differences in trainability. Indeed, our observed increases, (Irisin and SPARC), decreases (BDNF), and lack of change (Fractalkine and IL-6) in myokine concentrations were irrespective of adaptive phenotype. One potential explanation for the variation in change that we saw across our myokines of interest, may the impact of chronic exercise training on the endocrine system. Due to this impact, many target tissues increase their expression of hormone functional receptors, the hormone receptor affinity, and the cell post-receptor amplification mechanism (31), and this may explain the drop in post-training BDNF concentration observed herein. On the other hand, increases in Irisin and SPARC after a period of training could be driven by their positive role in metabolism, since both are proposed to enhanced glucose uptake and lipolysis (32–34) and demonstrate positive correlations to both BMI and fasting insulin (26, 35). In addition, the reduction of FGF21 in LRT animals is in line with previous studies where 3-wks of sprint interval training (36) and 3-months of combined resistance and aerobic training in obese woman (37) decreased FGF21 levels. However, Musclin remained elevated in LRT perhaps due to the low trainability of LRT and the lack of change in aerobic and metabolic status; instead, increases in Musclin in HRT fits with its putative role in exercise capacity (38).

### Impact of Acute Exercise on Circulating Myokines

An acute bout of exercise induces specific changes in circulating myokine concentrations (39) e.g. immediately with IL-6. However, the magnitude of the acute response, of IL-6 for example, has been linked to the intensity and duration of exercise, the number of muscles activated, and also the concentration of muscle glycogen (40). LRT/HRT animals were bred to show distinct adaptive responses, likely due to an accumulation of acute signalling and transcriptional events following each exercise bout (41). For example, a single exercise bout of similar intensity, confirmed by oxygen consumption and muscle glycogen concentrations, revealed contrasting transcriptional responses to exercise in LRT/HRT (11). However, hitherto nothing was known about the acute myokine response to a standardized exercise bout in these animals. Notably, acute myokine responses were not radically different between the LRT/HRT animals, which may indicate a limited role in adaptive capacity in relation to the contractile-induced release of myokines. Indeed, only Musclin showed a potential link to training capacity; in line with this, knocking out the Musclin gene leads to a decrease in aerobic capacity which was subsequently restored by recombinant Musclin administration (38). This may indicate a compensatory role of Musclin to improve low aerobic capacity in the LRT phenotype.

Upon investigation the impact of exercise training on acute responses to exercise, we were able to observe some clear differences with regard to the AUC of a number of myokines (i.e. compared to pre-training). Indeed, training significantly increased SPARC and Irisin concentrations, and reduced BDNF concentration in both LRT and HRT, while Fractalkine and Musclin yielded different responses to training across phenotypes. Our observation of an elevation of Fractalkine in HRT and not LRT animals was unexpected since elevated Fractalkine has been linked to obesity, insulin resistance and T2D (42). Nonetheless, Fractalkine is also proposed to block downstream TNF-α signalling (NF_K_B) and thus prevent insulin resistance in myotubes (43), indicative of a positive role in muscle. In addition, the correlation that we observed between the changes in AUC and change running distance, suggests a potential role of Fractalkine in exercise capacity; although further work is needed to establish this. That the acute temporal response of Musclin to acute exercise was increased after training only in the HRT animals, falls in line with the suggested role of Musclin in regulating aerobic capacity (38); a proposition supported herein by the tendency for a positive correlation with %Δ in running distance.

## Limitations and Conclusion

Firstly, we acknowledge the somewhat preliminary nature of these findings, in terms of total myokine coverage, and also where use of a multi-plex approach (while being efficient in terms of small volume tail vein sampling), may in instances yield less sensitivity than single-plexing. Also, as with studies of this nature, we may have missed temporal aspects e.g. the peak rise, in some myokines such as SPARC, Fractalkine, Irisin and BDNF which have been shown to peak ~30 min after acute exercise training (44, 45). We also accept that myokines may relate to adaptive traits that we haven’t captured. Nonetheless, our data are novel in demonstrating patterns of acute and chronic changes in plasma myokines in LRT/HRT rats. The LRT/HRT model develops gross heterogeneity in adaptive responses to exercise training that facilitates an understanding of extrinsic molecular networks responsible for such variation (4). Our data suggest a limited influence of training capacity in relation to myokines, but nonetheless, resting (baseline) levels of certain myokines may be influenced by metabolic traits induced by the out-breeding regime e.g. FGF21 and Musclin.

In sum, we conclude that: i) some baseline myokine abundance (FGF21, and Musclin) is linked to out-breeding polygenic genetic traits i.e. may be inherited, ii) myokines respond with temporal and quantitative specificity both to a single bout of exercise (Fractalkine, IL-6, SPARC, FGF21 and Musclin) and to sustained exercise training (Irisin, and SPARC), iii) there is limited evidence myokines can readily explain the marked differences in trainability of the LRT/HRT model. In sum, it is unlikely myokine responses can explain gross differences in trainability. Further studies, with specific and sensitive measurement approaches are needed to experimentally assess the physiological veracity of myokines role in adaptation to exercise training.

## Data Availability Statement

The original contributions presented in the study are included in the article/**Supplementary Material**. Further inquiries can be directed to the corresponding author.

## Ethics Statement

The animal study was reviewed and approved by The University Committee on Use and Care of Animals at University of Michigan.

## Author Contributions

All authors contributed to the article and approved the submitted version.

## Funding

This research was supported by the MRC-Versus Arthritis Centre for Musculoskeletal Ageing Research [grant numbers MR/R502364/1, MR/P021220/1] and the National Institute for Health Research (NIHR) Nottingham Biomedical Research Centre. The animal study conducted by the University of Michigan Animal Phenotyping Core was partially supported by P30 grants DK020572 (MDRC) and DK089503 (MNORC) [Funding: Grant P40OD021331 to LGK and SLB from the National Institutes of Health]. Contact LK (Lauren.Koch2@UToledo.edu) or SB (brittons@umich.edu) for information on the LRT and HRT rats: these rat models are maintained as part of an Exercise Rat Resource for Researchers (ER3) at the Centre for Hypertension and Precision Medicine, The University of Toledo, Toledo, Ohio. WF was supported by a government PhD studentship grant of Umm Al-Qura University, Makkah, Saudi Arabia.

## Conflict of Interest

The authors declare that the research was conducted in the absence of any commercial or financial relationships that could be construed as a potential conflict of interest.

## Supplementary Material

Supplementary Figure 1 Change in animal’s body weight (grams) in low response trainers (LRT) and high response trainers (HRT). Values are means±SD. Analysis via two-way ANOVA with Sidak’s post hoc test. Main effects are shown in the text box **P<0.01, ***P<0.001, ****P<0.0001. Non-underlined symbols represent within group differences. Underlined symbols represent between group differences.

## Abbreviations

LRT, Low response trainer; HRT, High response trainer.
