# Supplementary figures and images for: Myokine Responses to Exercise in a Rat Model of Low/High Adaptive Potential
Source: Front Endocrinol (Lausanne). 2021 Jun 9;12:645881. doi: 10.3389/fendo.2021.645881 (PMC8220071; doi:10.3389/fendo.2021.645881)

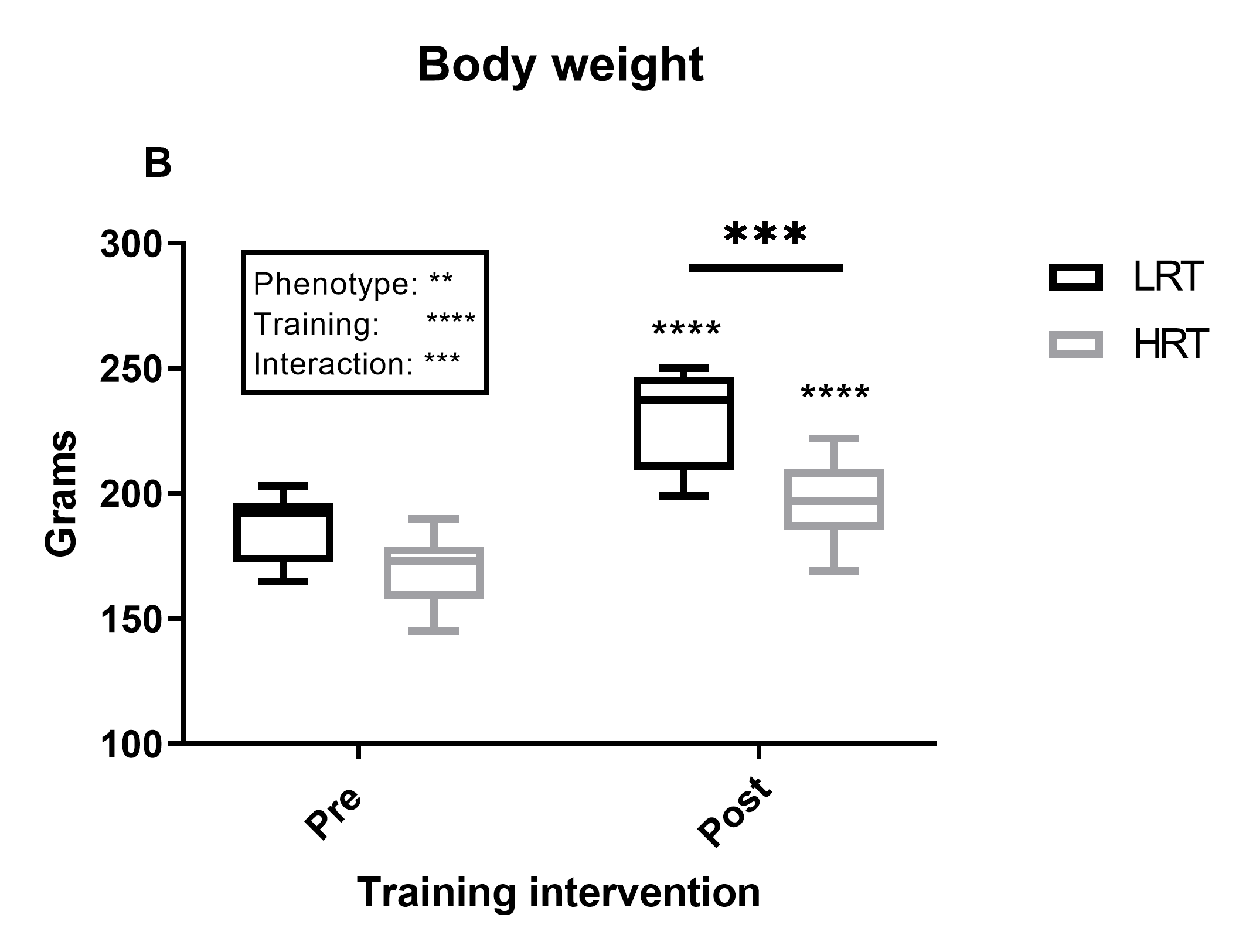

Supplement: Supplementary Figure 1 — Change in animal’s body weight (grams) in low response trainers (LRT) and high response trainers (HRT). Values are means±SD. Analysis via two-way ANOVA with Sidak’s post hoc test. Main effects are shown in the text box **P<0.01, ***P<0.001, ****P<0.0001. Non-underlined symbols represent within group differences. Underlined symbols represent between group differences. [file Image_1.tif]
