# Supplementary material for: Myokine Responses to Exercise in a Rat Model of Low/High Adaptive Potential
Source: Front Endocrinol (Lausanne). 2021 Jun 9;12:645881. doi: 10.3389/fendo.2021.645881 (PMC8220071; doi:10.3389/fendo.2021.645881)
Supplement: Supplementary file 2 [file Table_1.pdf]

Table 1. Myokine sensitivity multiplex ELISA. Minimum detection level (Min DC) of 3 assays.

Analyte	Min DC (3 assays)	Standard range
BDNF (pg/mL)	3.56	7-30,000
Erythropoietin (pg/mL)	3.99	24-100,000
IL-15 (ng/mL)	0.14	0.5-2000
FGF21 (pg/mL)	7.01	5-20,000
Fractalkine (pg/mL)	1.60	5-20,000
IL-6 (ng/mL)	0.17	0.2-1000
FSTL-1 (pg/mL)	29.29	98-400,000
GDF8/Myostatin (ng/mL)	0.32	1-4000
Irisin (ng/mL)	0.28	0.5-2000
LIF (pg/mL)	13.36	49-200.000
Osteocrin (pg/mL)	5.66	10-40,000
SPARC (ng/mL)	0.13	0.2-1000
